# Predictors That Identify Complications Such As Anastomotic Leak in Colorectal Surgery: A Systematic Review

**DOI:** 10.7759/cureus.28894

**Published:** 2022-09-07

**Authors:** Tharun Yadhav Selvamani, Shoukrie I Shoukrie, Jyothirmai Malla, Sathish Venugopal, Ramaneshwar Selvaraj, Ravneet K Dhanoa, Anam Zahra, Ranim K Hamouda, Aishwarya Raman, Jihan Mostafa

**Affiliations:** 1 General Surgery, California Institute of Behavioral Neurosciences & Psychology, Fairfield, USA; 2 Orthopaedics and Traumatology, California Institute of Behavioral Neurosciences & Psychology, Fairfield, USA; 3 Internal Medicine, California Institute of Behavioral Neurosciences & Psychology, Fairfield, USA; 4 Neurology, California Institute of Behavioral Neurosciences & Psychology, Fairfield, USA; 5 Internal Medicine/Family Medicine/General Surgery, California Institute of Behavioral Neurosciences & Psychology, Fairfield, USA; 6 Surgery, California Institute of Behavioral Neurosciences & Psychology, Fairfield, USA

**Keywords:** anus, rectum, colorectal cancer, colorectal surgery, complications, anastomotic leak, inflammatory marker, white cell count, procalcitonin, c reactive protein

## Abstract

Leakage of the anastomotic site is considered to be one of the most serious complications after colon and rectal surgery. It is associated with increased mortality, morbidity, and longer hospital stays. This systematic review examines the need for blood markers such as C-reactive protein (CRP), procalcitonin (PCT), albumin, and various other molecular markers that assist in their propensity to diagnose anastomotic leakage (AL) early after surgery.

Utilizing PubMed and Google Scholar as resources and including the Preferred Reporting Items for Systematic Reviews and Meta-Analyses (PRISMA) guidelines for the articles, and studies over the last five years were included. A total of 12 studies have been discussed, and most articles suggest CRP as an excellent indicator. CRP compared to Dutch leakage scores (DLS) and PCT studies suggest that the three combinations improve the predictable outcome of AL. In addition, CRP and PCT have been shown to diagnose AL early in the postoperative period. Other studies include the role of markers of oxidative stress markers, Interleukin-6, Interleukin-10, and other molecular markers in the peritoneal drain which are predictive for identifying AL after three days postoperatively (POD-3).

Overall, CRP has proven to be a reliable standard indicator of diagnosis. This is because the postoperative elevation of this protein indicates a problem of leakage with clinical symptoms. Other blood parameters are useful for diagnosis as well, but the limitations are the lack of appropriate studies and the number of randomized controlled trials in this area of ​​study.

## Introduction and background

Leakage of the anastomotic site is one of the life-threatening complications after colon and rectal surgery. According to a recent study by the International Research Group for Rectal Cancer, an anastomotic leakage (AL) is defined as a defect in the intestinal wall of the anastomotic site including the sutures and staple lines of the neo rectal reservoir, intraluminal and extra luminal compartments [1]. European Society of Coloproctology (ESCP) snapshot audit in 2015 found that 8.1% of patients developed AL after right hemicolectomy [2]. Any gastrointestinal anastomosis can lead to an increased risk of mortality, morbidity, hospitalization, and associated hospital costs [3]. Clinical manifestations of AL are abdominal pain, tachycardia, dynamic ileus, and swinging pyrexia leading to peritonitis, abscess formation, and sepsis [4]. The aetiology of AL is multifactorial, depending on whether it is preoperative or intraoperative, such as emergencies, prolonged surgery, and increased body mass index (BMI) [5].

Colorectal cancer (CRC) is the third most common type of cancer in the world. The significant increase in the number of people suffering from colon cancer in Western countries is due to the increased consumption of animal-derived meat and fat [6]. The latest treatments for primary and metastatic CRC are laparoscopic surgery for the primary disease followed by resection of the metastatic disease, radiation therapy, neoadjuvant chemotherapy, and palliative chemotherapy [7]. Treatment of CRC is based on the location of the tumour and the lymph nodes involved [8]. If screening tests for CRC such as the faecal immunochemical test of stools are done and it comes positive, the next best step is to perform a colonoscopy of the patient.

The surveillance and screening of CRC by colonoscopy have shown the importance of early detection of cancers [9]. Changes in perioperative surveillance and surgical techniques have had important consequences for reduced postoperative mortality [10]. Early detection of leakage at the anastomotic site helps in the early detection, treatment, and prevention of post operative complications, sepsis, and mortality [11]. There are different strategies for identifying AL using different markers, including CRP and WCC [12]. CRP is being studied as a specific early protein marker for postoperative complications [13]. Acute phase reactants are produced by hepatocytes in response to inflammatory cytokines. The tendency for CRP usually increases 48 hours after surgery. A steady trend showing increased inflammatory markers would suggest looking out for an AL with the clinical features [14].

## Review

The need for systematic review

While further studies are underway, many studies have shown that the role of postoperative CRP and trajectory is an excellent diagnostic indicator for eliminating complications such as AL. CRP has a high negative predictive value (NPV). This is a reminder that values ​​below the cut-off level are unlikely to indicate AL. The importance of early diagnosis is to prevent associated mortality and morbidity. Other parameters help identify potential complications, such as the role of oxidative stress markers, WCC, procalcitonin (PCT), albumin, and molecular markers. However, since CRP is a good predictor of leak detection, the answer to the question of which markers to use to detect AL may be the most accurate.

Methods

A literature search for this systematic review was performed on databases such as PubMed, Google Scholar, Cochrane, and ScienceDirect. We also adhere to the Preferred Reporting Items for Systematic Reviews and Meta-Analyses (PRISMA) Guidelines. The search for the articles was started on June 10, 2022 using the keywords “C reactive protein OR Inflammatory marker OR Globulin AND Anastomotic leak OR Complications OR Channel leak AND Colorectal surgery OR Cancer surgeries OR Rectum OR Anus OR Colon AND Predict OR Indicator OR Estimate.”

Several studies that included patients who had anastomotic leak following colorectal surgeries studies and the role of inflammatory markers such as C-reactive protein (CRP) to predict the complications were included in our search. Using the MeSH keywords and phrases (“C-Reactive Protein/blood” [Majr] OR “C-Reactive Protein/therapeutic use” [Majr] ) AND (“Anastomotic Leak/analysis” [Majr] OR “Anastomotic Leak/blood” [Majr] OR “Anastomotic Leak/diagnosis” [Majr] OR “Anastomotic Leak/statistics and numerical data” [Majr] OR “Anastomotic Leak/surgery” [Majr]) AND (“Colorectal Surgery/classification” [Majr] OR “Colorectal Surgery/mortality” [Majr] OR “Colorectal Surgery/surgery” [Majr]) AND (“Forecasting/methods” [Mesh] OR “Forecasting/standards” [Mesh] ). Out of which, 85 studies that were relevant to the study were found in the various databases.

**Figure 1:**
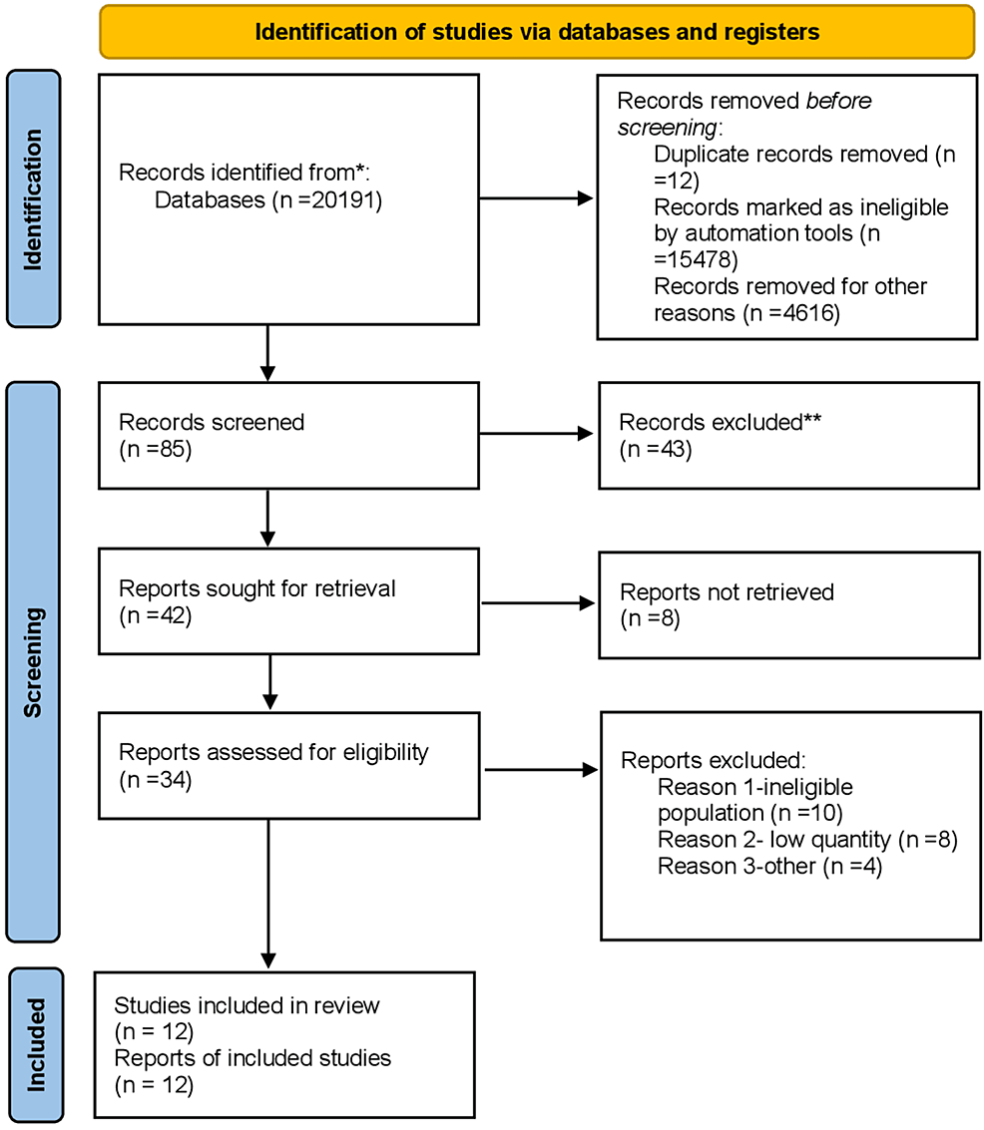
PRISMA flow diagram

A total of 20,191 studies were found on PubMed, and Google Scholar even before the application of any inclusion or exclusion criteria. After the application of the inclusion, exclusion criteria, removal of duplicates, articles within the last five years, and the title of the article were used for the final selection. All the selected articles underwent quality appraisal by the application of the Assessment of Multiple Systematic Reviews (AMSTAR) checklist and the Newcastle-Ottawa Quality Assessment Scale, which came up to 12 studies. Figure 1 shows the PRISMA flow diagram detailing the study selection process.

Results

A total of 12 studies involving different types of studies determining the ability of the inflammatory markers of CRP, WCC, PCT, and different markers were compared to see if they are effective in diagnosing AL. Certain studies show why CRP is considered a higher standard than markers. CRP has consistently been shown to be an excellent indicator for the diagnosis of anastomosis after colorectal surgery by measuring daily postoperative values, whereas one of the studies that compared CRP with PCT and WCC showed very similar trajectories after surgery. The characteristics and features of AL are grouped into a clinical score called as Dutch Leakage Score (DLS), which has been found as an essential tool for diagnosing and treatment. DLS and CRP are excellent predictors for AL on addition PCT has shown that increased the predictive value for identifying AL. In addition, in other studies, the other risk factors for developing AL after surgery have been investigated. These suggest different factors that are important in the development of leaks, and among the male gender, the site of anastomosis, and especially lower anastomosis were found to be higher risk factors. These aetiological factors must be studied in detail to better understand the cause.

Furthermore, a study performed showed that the expression patterns of inflammatory markers in serum and peritoneal fluid from the abdominal drain on a postoperative day three (POD-3) such as leukaemia inhibitory factor (LIF), IL-16 (interleukin-16), and IL-21 (interleukin-21), CXCL5/ENA-78 (C-X-C motif chemokine 5 or epithelial neutrophil-activating peptide), CCL-1/I-309 (C-C motif ligand 1 or inflammatory cytokine I-309), CCL8/MCP-2 (Chemokine C-C motif ligand 8 or monocyte chemotactic protein-2), CCL13/MCP-4 (Chemokine C-X-C motif ligand 13 or monocyte chemotactic protein-4) were selected for analysis. The levels of CCL8/MCP2, LIF, and CXCL5/ENA-78 were found to be increased in patients with AL on POD-3 after colorectal surgery.

Another study was done on the role of oxidative stress markers in diagnosing leaks which studied both peritoneal fluid and systemic blood and compared the trajectories in careful diagnosing of AL. It said the combination of both these values would be an excellent indicator for diagnosing an AL rather than isolated blood measurements. Finally, a retrospective study was performed on CRP and its role in identifying serial AL after laparoscopic transabdominal excision. The levels specifically on POD-3 and POD-7 need careful evaluation as they could be the predictor of the leaks. The characteristics of the studies used for this review are included in Table 1.

**Table 1:** Description of the studies that met the inclusion criteria for this review DLS: Dutch Leakage Score, CRP: C-Reactive Protein, PCT: Procalcitonin, AL: Anastomotic Leak, LAR-TME: Low Anterior Resection -Total Mesorectal Excision, POD: Post Operative Day, CCL8/MCP-2: Chemokine C-C motif ligand 8/Monocyte Chemotactic Protein-2, LIF: Leukotriene Inhibitory Factor, CXCL5/ENA-78: C-X-C motif chemokine 5/Epithelial Neutrophil -Activating Peptide, WCC: White Cell Count, BT: Body Temperature, CRC: Colorectal Cancer

Author	Purpose of the study	Study type	Sample Size	Main findings
Jin et al. [15]	CRP determination is an excellent predictor of anastomotic leakage in laparoscopic transabdominal rectal resection.	Retrospective Single Centre	196	CRP is a reliable predictor of anastomosis after laparoscopic transabdominal resection. Elevated CRP levels on POD (4-7) suggest the need for more careful patient evaluation.
Messias et al. [16]	Serum CRP is a useful marker to rule out AL after colon and rectal surgery.	Prospective Cohort Study	90	Postoperative serum CRP levels in patients undergoing colorectal surgery with primary anastomosis may become a useful marker to rule out AL.
Amoli et al. [17]	The role of serum CRP, WCC, BT in the detection of AL, and the value of postoperative CRP level in excluding AL.	Retrospective	315	Postoperative serum CRP levels of 44 mg / L and 27.2 mg / L, especially on days two and four, maybe early and sensitive markers for the exclusion of AL.
Reynolds et al. [18]	The ability of CRP to predict AL in the first week after anterior resection for rectal cancer is significant.	Retrospective	211	On the POD-5, the CRP value was one hundred and thirty two, the sensitivity was 70%, and the specificity was 76.6%. Using CRP as a test helps rule out the development of AL after anterior resection.
El Zaher et al. [19]	Investigate the role of the triad PCT, CRP and WCC trajectories as a predictive biomarker for the AL after colorectal surgery.	Prospective cross-sectional study	217	CRP, PCT, and WCC trajectories of combined measurements had a better predictive power for AL than the isolated daily measurements.
Italian ColoRectal Anastomotic Leakage (iCral) Study Group et al. [20]	Use of the DLS, serum CRP and serum PCT in the diagnosis of AL after elective colorectal resection.	Prospective Multi centre Observational Study	1546	DLS and CRP levels are good positive predictors and excellent negative predictors of AL; The addition of PCT improved the predictive value for the diagnosis of AL.
Chernyshov et al. [21]	Examine the factors involved in the AL following low anterior resection and total mesorectal excision (LAR-TME) and to determine the usefulness of early measurement of the inflammatory biomarkers CRP and PCT.	Retrospective Cohort Study	100	To examine factors associated with AL to determine the utility of early measurement of inflammatory biomarkers CRP and PCT
Shimura et al. [22]	The association between perioperative albumin levels and AL has not been fully investigated in patients with Colorectal cancers.	Retrospective study	200	Postoperative hypoalbuminemia in patients undergoing colon and rectal surgery is a possible sign of AL.
Luo et al. [23]	Oxidative stress level is a predictor of anastomotic leakage after rectal surgery.	Retrospective	270	The oxidative stress indicators we tested have the potential to be used to diagnose an AL in serum and drain fluid.
Sua et al. [24]	Serum CRP levels, especially on days 2 and 4 after surgery, could be an indicator of serious infection. If this level falls below a certain cut-off, it might be an indication that there is an infection present.	Systematic review	36	Evaluated fifty one different biomarkers after surgery. The most commonly evaluated from peritoneal drainage fluid were IL-6, IL-10, and tumour necrosis factor. Systemic markers included CRP, WCC, and PCT. The combination of markers has improved the effectiveness of leak detection.
Klupp et al. [25]	The importance of a particular protein expression profile as a putative bio maker for AL was investigated.	Prospective cohort study-Single centre	270	Evaluated fifty one different biomarkers after surgery. The most commonly evaluated from peritoneal drainage fluid were IL-6, IL-10, and tumour necrosis factor. Systemic markers included CRP, WCC, and procalcitonin. The combination of markers has improved the effectiveness of leak detection.
Sciuto et al. [26]	Predictor of anastomotic leakage after laparoscopic colon and rectal surgery.	Systematic review	-	Adequate knowledge of the risk factors is essential to identify high-risk patients and select them correctly for stoma deviation in order to minimize the serious clinical consequences of anastomosis.

Discussion

Role of CRP to Predict Anastomotic Leak

A retrospective study conducted by Sir Run Shaw Hospital included 196 patients with rectal cancer [15]. These patients underwent a procedure called laparoscopic transabdominal rectal resection without ileostomy. The various parameters included were patient details, complication symptoms, CRP levels, and neutrophils assessed by post operative day 7. The results showed that about 11 of the 186 patients had AL. It was stated that CRP levels from POD (4-7) after surgery were found to be reliable in detecting AL. Additionally, it was concluded that monitoring CRP levels would help earlier management planning.

Another retrospective study was conducted by Messias et al. Ninety subjects participated in Carapicuiba General Hospital for four years. All of these patients underwent emergency or elective colon and rectal surgery with anastomosis [16]. In these patients, postoperative CRP levels were measured from POD (1-7). The drop in CRP values after POD-2 is unaffected by factors such as individual inflammatory response, type of approach, or surgical indication, thus ruling out AL. We also concluded that CRP levels on POD-4 are the most effective indicator. The study also mentioned that CRP, gradually over the next few postoperative days and was less than 180 milligrams per litre (180 mg/L) on POD-4. This is a mandatory criterion for patient discharge due to the low probability of AL.

A retrospective study by Amoli et al. was conducted for more than two years including 315 patients. All patients underwent elective bowel surgery, and their CRP levels were measured five days after surgery to rule out complications such as AL [17]. Symptoms of AL include fever, increased WCC count, body temperature, increased reactive protein levels, and intestinal obstruction. Approximately 26 patients had AL. They concluded that the CRP was highly sensitive, and it reduced hospitalization on the POD from 2 to 4.

A retrospective study by Reynolds et al. was conducted for five years. The study included 211 patients. 70% of postoperative CRP levels were examined on days 5, 6, and 7. In addition, these elevated CRP levels are associated with an increased risk of AL. It also helps to detect AL early to minimise postoperative complications [18].

Other Predictors to Estimate the Anastomotic Leak

A study by El Zaher et al. was done over two years and included 205 patients with an average age of 56 years. This 2020 study focused primarily on the role of PCT, CRP, and WCC in 10.7% of patients. This study demonstrated that PCT is the best predictor of AL compared to WCC and CRP on POD-5. In addition, a five-day combined measurement of WCC, CRP, and PCT was concluded to be an ideal predictor of AL. They also suggested that these levels are essential for the early discharge of patients and improved recovery programs [19].

The Italian Colorectal Anastomotic Study Group (iCRAL) conducted a one-year multicentre prospective observational study in Italy back in 2017 which included 1,546 patients from a total of 2,717 resections. Patients who had a stoma before or during surgery, emergency treatment, or patients who closed the stoma without resection were excluded. Of these, 4.9% had an anastomotic leak. The DLS is a specific diagnostic and therapeutic algorithm that provides a score for signs and symptoms of an anastomotic leak. CRP level showed good prediction on POD-6. In addition, the roles of CRP, PCT, and DLS had the overall effect on AL, with the highest predicted value of POD-6 at 47.1%. It was concluded that CRP and DLS are excellent negative predictors and support the role of PCT in diagnosis [20].

A retrospective study by Chernyshov et al. included a hundred patients who participated for three years. Of those who underwent deep anterior resection and total mesenteric resection with proximal drainage stoma, approximately 11 clinical leaks, and was found that perioperative transfusion was an independent risk factor for AL. CRP levels were found to be high on POD-3 and POD-6. From this, we can conclude that the high CRP and PCT levels had high negative predictions on both days [21].

A retrospective study by Shimura et al. included 200 patients who underwent curative laparoscopic surgery for CRC. Perioperative albumin levels and various other markers were evaluated to find an association with AL. Complications occurred in 5.6% of patients and more frequently in patients with rectal cancer. This study suggested that the role of postoperative hypoalbuminemia may be a strong predictor of AL, even in the absence of clinical signs. This is useful for treatment if the patient maintains nil by mouth (NBM) or has surgery to prevent complications [22].

Role of Oxidative Stress and Inflammatory Biomarkers in Diagnosing the Anastomotic Leak

In addition to CRP, markers of oxidative stress have been shown to help diagnose AL after colon and rectal surgery. Reactive oxygen species (ROS) are required for microbial control and cell signalling, but when present at higher levels than necessary, they can cause tissue damage and necrosis. In addition, oxidative stress has been reported to increase matrix metalloproteinases (MMPs) that may be involved in the breakdown of collagen, which leads to tissue necrosis. This is evidenced by a decrease in the antioxidant markers catalase (CAT) and superoxide dismutase (SOD), and an increase in malondialdehyde (MDA), which is responsible for lipid peroxidation and denaturation [23].

A prospective study was conducted in 2021 and all patients diagnosed with rectal cancer underwent elective rectal surgery with a primary anastomosis. The data analysed showed oxidative stress markers such as CAT, MDA, and SOD in serum and drain fluid. The three redox indicators are more important in diagnosing AL of serum and drain compared to CRP and neutrophil percent. This study concludes that these levels of oxidative stress are reliable indicators, and in particular MDA is comparable to CRP, demonstrating its usefulness in diagnosing AL [23].

A systematic review was conducted by Su'a et al. to assess biomarkers as potential diagnostic tests for preclinical detection of AL. Of these 31 studies, 51 different biomarkers were evaluated in the context of AL after colorectal surgery. The systemic and peritoneal levels of Interleukin-6 (IL-6), Interleukin-10 (IL-10), and Tumour necrosis factor (TNF) were evaluated. IL-6 and TNF were found to be elevated early postoperatively in predicting AL. Systemic blood showed CRP, leukocytes, and PCT, and the interfering combination of peritoneal and systemic blood showed increased efficacy rather than isolated blood [24].

A prospective cohort study by Klupp et al. was done at the University of Heidelberg in Germany on serum and peritoneal drain samples collected on the POD-3 [25]. A total of 38 patients who underwent colorectal surgeries were analysed and out of which eighteen had an AL. The samples from the drain were analysed using an enzyme-linked immunosorbent assay (ELISA) and laboratory analysis. There was a panel of inflammatory markers including CCL13/MCP-4 (monocyte chemotactic protein-4), which carries out pro-inflammatory actions through chemotaxis of macrophages, basophils, and lymphocytes. LIF aids the recruitment of cells to the damaged areas, IL-21 (interleukin-21) expression is induced by other cytokines. IL-16 (interleukin-16) is produced by T-lymphocytes, eosinophils, and mast cells and can be recruited at the time of cell necrosis. CCL-1/I-309 (C-C motif ligand 1) is produced by regulatory T cells in areas of inflammation, and CCL8/MCP-2 (monocyte chemotactic protein-2) activates natural killer cells as a pro-inflammatory mediator. CXCL5/ENA-78 (epithelial neutrophil-activating peptide) is located in the inflamed intestinal mucosa. This study concluded that the inflammatory markers such as CCL8/MCP-2, CXCL5/ENA-78, and LIF were increased on POD-3 and hence could be potential markers for the detection of AL.

Causes for Anastomotic Leak Following Colorectal Surgery

A systematic review was conducted in 2018 to understand the risk factors for AL after colorectal surgery. It has been found to vary from 0% to 20% with a significant reduction compared to the laparoscopic approach. When they examined the risk factors for left anastomosis, we found that the risk factors were low at the anastomotic site of male patients. It also shows that the role of microorganisms is a risk factor for leakage. The study concluded that the role of diverting stoma was considered to analyse the association between leaks and predictors [26]. There are many possible aetiology for an AL, depending on the site. These can include right-sided, left-sided, and intra-corporeal anastomoses. A retrospective study found that out of 423 patients who underwent laparoscopic colonic resection and anastomosis, the leak rate was around 3.78% [27].

Smoking was found to be a cause of vascular ischemia and carbon monoxide cellular hypoxia, which would impair anastomotic circulation [28]. Many patient-related factors can affect anastomotic healing, such as male sex, increased BMI, and preoperative nutritional status. Tumour size and stage, postoperative hypoalbuminemia, neoadjuvant therapy, and postoperative diarrhoea were found to be associated with AL.

Limitations

The primary weakness noted in this study included a lack of adequate sample size and hence meta-analysis would not hold any benefit. Assessment of a larger sample size over a longer period would lead to a more beneficial study and an increase in accuracy. Other limitations are the absence of randomized control trials in this area of research.

## Conclusions

Overall, a myriad of research studies shows the efficacy of CRP and how it remains one of the most reliable markers in comparison to the rest. However, in comparison to CRP, other parameters including oxidative stress levels, PCT, and WCC show also promising results and are being used quite recently used markers than the former. In addition, this study also highlights the usefulness of the combined triad of PCT, CRP, and WCC trajectories as accurate biomarkers for AL after colorectal surgery. Furthermore, this combination can be a reliable predictor for early patient discharge, which would be highly beneficial to enhanced recovery after surgery programs. Lastly, this study concludes by emphasizing conducting research over larger study groups with long periods for a better understanding of the advantages and disadvantages of CRP in comparison to other predictors of AL.
